# Assessment of Shape Changes of Mistletoe Berries: A New Software Approach to Automatize the Parameterization of Path Curve Shaped Contours

**DOI:** 10.1371/journal.pone.0060522

**Published:** 2013-04-02

**Authors:** Renatus Derbidge, Linus Feiten, Oliver Conradt, Peter Heusser, Stephan Baumgartner

**Affiliations:** 1 Centre for Integrative Medicine, University of Witten/Herdecke, Witten, Germany; 2 Research Institute at the Goetheanum, Science Section, Dornach, Switzerland; 3 Faculty of Engineering, Chair of Computer Architecture, University of Freiburg, Freiburg, Germany; 4 Section for Mathematics and Astronomy, Goetheanum, Dornach, Switzerland; 5 Hiscia Institute, Society for Cancer Research, Arlesheim, Switzerland; Pennsylvania State University, United States of America

## Abstract

Photographs of mistletoe (*Viscum album* L.) berries taken by a permanently fixed camera during their development in autumn were subjected to an outline shape analysis by fitting path curves using a mathematical algorithm from projective geometry. During growth and maturation processes the shape of mistletoe berries can be described by a set of such path curves, making it possible to extract changes of shape using one parameter called Lambda. Lambda describes the outline shape of a path curve. Here we present methods and software to capture and measure these changes of form over time. The present paper describes the software used to automatize a number of tasks including contour recognition, optimization of fitting the contour via hill-climbing, derivation of the path curves, computation of Lambda and blinding the pictures for the operator. The validity of the program is demonstrated by results from three independent measurements showing circadian rhythm in mistletoe berries. The program is available as open source and will be applied in a project to analyze the chronobiology of shape in mistletoe berries and the buds of their host trees.

## Introduction

Patterns and forms in animals and plants have fascinated mankind from the dawn of science in ancient Greece, and motivated thinkers and scientists at the beginning of the scientific revolution. Patterns and forms seemed ideal for exploring the hidden plan of God in nature [1], with mathematics the tool of choice for revealing these “divine thoughts”. Even today, in fact, amazing correlations continue to be found between forms and patterns in nature and certain fields in mathematics [2], and modeling has developed into a viable instrument for revealing the lawfulness of biological processes.

In chronobiology, mathematics and biology interact in a peculiar way. Rhythms of plant growth and development, the synchronicity of cell division and DNA replication are scrutinized, as are gene expression and enzymatic activity in correlation with time or season. Biological rhythms can also be found phenotypically in plant organs such as buds or berries [3].

Lawrence Edwards, a Scottish mathematician, discovered that two-dimensional projections of a certain subgroup of path curves [4], [5], [6], which are three-dimensional transformations of measures in projective geometry [4], [5], [6], [7], fit perfectly to the outline shape of various plant buds [8]. Other researchers observed that the profile of certain berries (e.g. of mistletoe, *Viscum album* L.) likewise accord with path curves [9], [10], [11]. Edwards observed that the shape of buds of various trees during dormancy in winter exhibited a fortnightly rhythmic change of form [8]. One of many path curve-defining parameters called Lambda (λ) is needed to determine the change of shape. λ is of interest, since it defines the outline shape of a path curve. Lambda allows the detection of possible alterations of shape in time.

We aimed to investigate the reproducibility of Edwards's results, and to extend his approach to shorter timescales. This requires the assessment of a large number of plant photographs, taken at short intervals over a period of several weeks or even months. Form analysis of buds or berries on the basis of such pictures evidently requires software to automatize the process. Such software must facilitate determination of the two-dimensional outline shape of buds or berries.

The outline shape of a three-dimensional path curve, which is defined by the parameter λ can be deduced from the contour line of the object captured in the photograph. The software recognizes the outline as a contour line and extracts λ by calculating the path curve that best fits and corresponds to the outline shape of the object.

Since some of the procedures require an operator's deliberate actions, the pictures must be analyzed in a random order and without any knowledge of the time and day they were taken. To our knowledge, appropriate software capable of performing these tasks does not exist. Thus, we decided to design and implement a program according to the specifications outlined above.

## Results

The software developed is called “LambdaFit” and is currently available as version 1.7. The program is freely available as open source at http://www.feiten.de/LambdaFit/. Its main features are described in the following paragraphs.

### Import, randomization and coding of the images

The human operator of the software must not know which picture he is currently evaluating. Otherwise he might subconsciously bias the measurements. Therefore, the first task of LambdaFit is to code a set of images.

**Figure 1:**
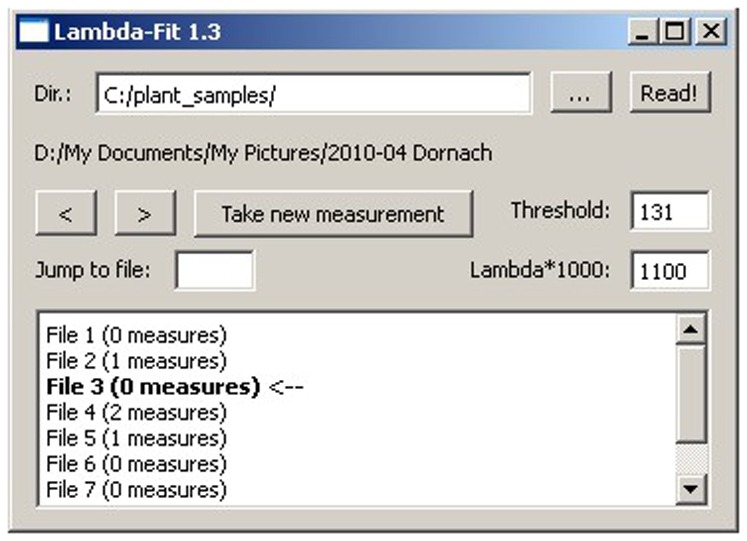
Graphic user interface. In this case the original files from the folder “plant_samples” are shown in random order. Files 2 and 5 have been measured once, file 4 has been measured twice, and the other files shown have not been measured yet. File 3 is selected. The values of the fitted parameters are saved automatically in a file not visible in this interface in the same folder as a “csv”-file.

The software prompts the operator to select a directory of images, which are subsequently sorted in random order and listed for the operator with coded numbers only (see Figure 1). In the same directory, a file is created which stores this random order. Should a new order be needed, it suffices to delete the first file.

The operator looking at the interface of the software only sees the number of measurements performed for a given set of images. Thus, it is possible to repeat measurements for one and the same folder in different random orders, and without the risk of inadvertently missing a picture (see Figure 1).

The software's graphic user interface has some features to make use as convenient as possible. After opening a folder of photographs (JPEGs) the feature “<” and “>” lets the operator skip from file to file, shown in the bottom text box. The “Jump to file” text box can be used to directly select a specific file. “Take new measurement” opens the windows to determine the best path curve parameters. The optimization process can then begin (see below). Further features give the opportunity to change some parameters of the path curve, which are pre-set as shown in the interface box. The text fields “Threshold” define the value for contour recognition. This is the value indicating the lightness or darkness of the threshold to decide if a pixel will be defined as black or white. The box “Lambda*1000” can be used to define the start value of λ, as buds and berries, for instance, have a quite different λ-value. Definition of those two parameters will be saved for the “Take new measurement” part, and stay the same until changed again.

### Recognition of the plant contour

In order to compute the parameters of the path curve with best fit to the biological sample, the software must first facilitate shape identification. Free graphics library openCV [http://opencv.willowgarage.com/] is very well suited for this task, since it already provides many computer vision functionalities, such as recognition of contours in images [12].

**Figure 2:**
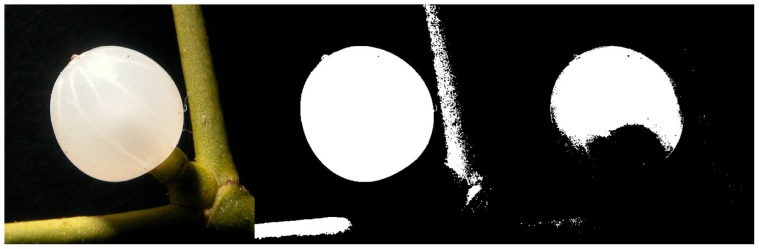
Drawing the contour (threshold function). Original photograph of a mistletoe berry (left) with two binary conversions: with adequate threshold (center), with inadequate threshold (right).

In order to detect contours in a picture, the latter first has to be converted into a binary image composed exclusively of black and white pixels. The threshold level must be set by a human operator, based on the picture's lighting. Figure 2 shows different outcomes for different threshold values.

**Figure 3:**
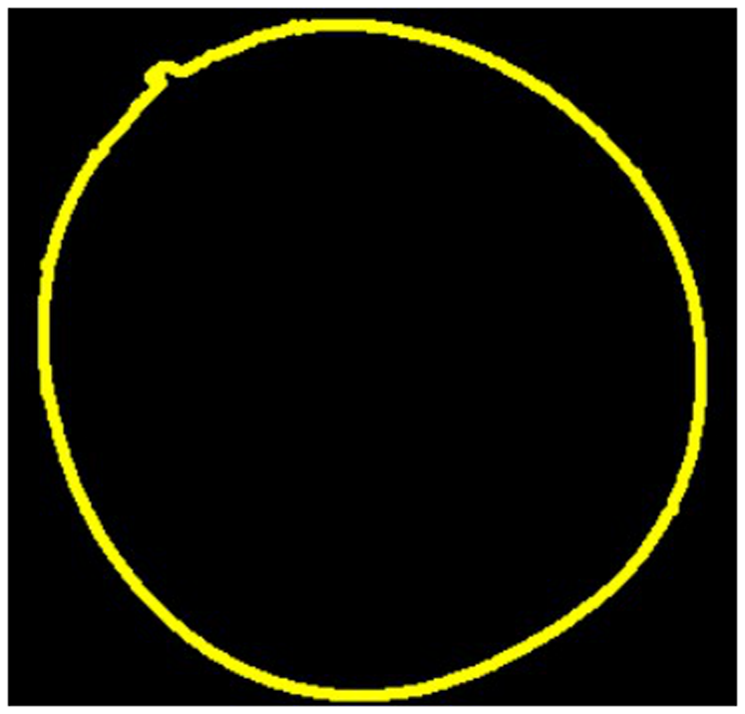
Contour line. The contour of the mistletoe berry in Fig. 2, as determined by LambdaFit.

It is very important that the outline of the photographed object is clearly silhouetted against the background and not blurred. Varying thresholds lead to different shapes determined by LambdaFit. Based on the binary image (middle) in Figure 2, the software is able to derive the sample's contour as depicted in Figure 3.

### Drawing the path curve

**Figure 4:**
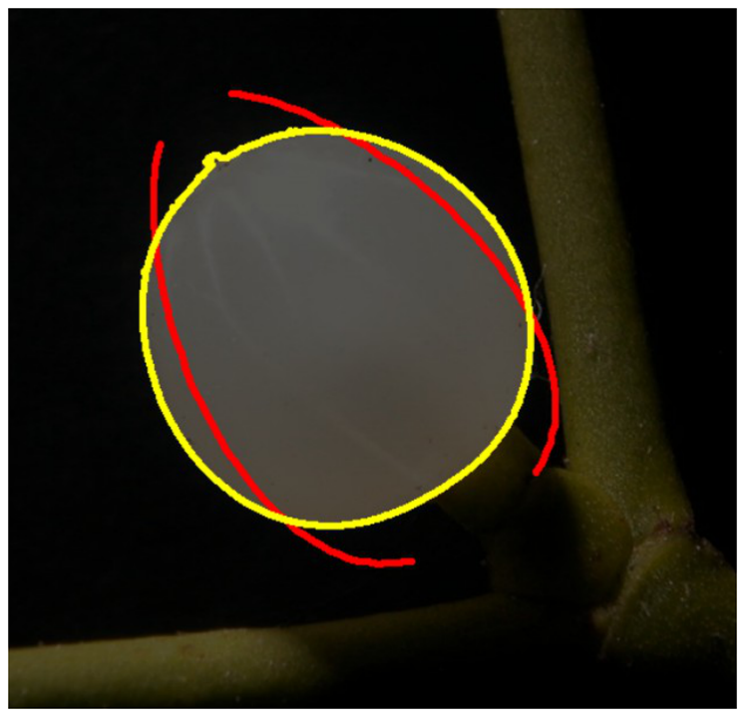
Contour and calculation line for the path curve. Composite picture of a mistletoe berry with the contour in yellow and the calculated – not yet fitted – path curve in red.

**Figure 5:**
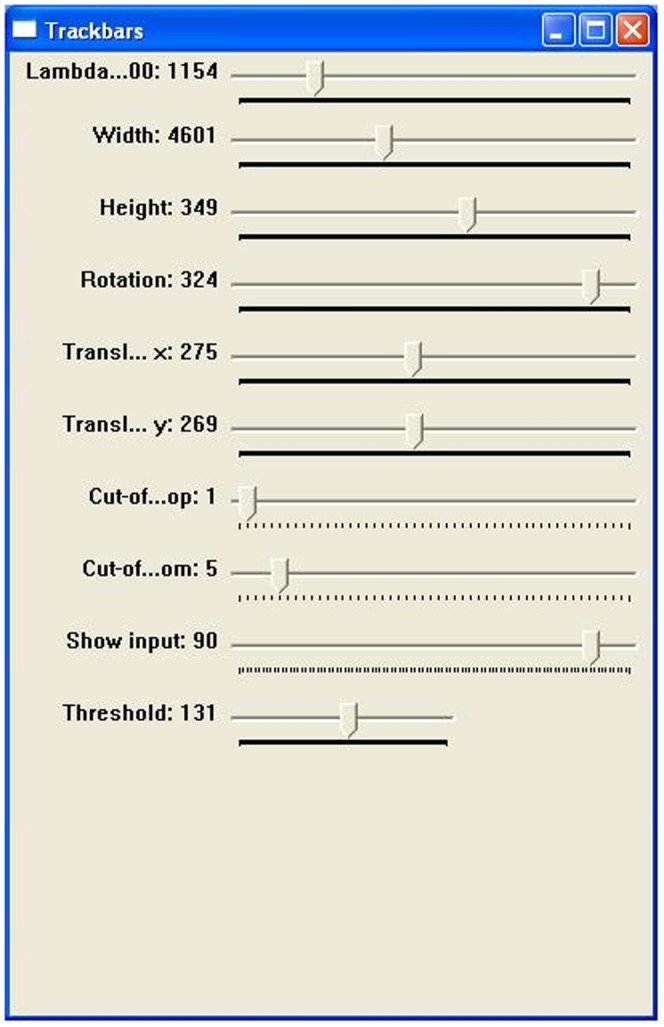
“Trackbar” window. All necessary parameters are shown in an additional window that contains slides for manual adaptation of the parameters of the path curve to the contour.

The software calculates and draws a complete path curve, whose starting position, shape and orientation can be adjusted by changing a set of parameters (see below). In a second step, the parameter values can be deduced for the best fitting path curve, i.e. that of maximum congruency. Details of the algorithm used and the optimization process are described in the section “Design and Implementation”. Figure 4 shows the appearance of a picture at the stage of measurement as described above. The drawn path curve is visible for the user as a red line according with the different parameters that define the shape and the position of the path curve outline (see Figure 5).

### Extraction of Lambda

**Figure 6:**
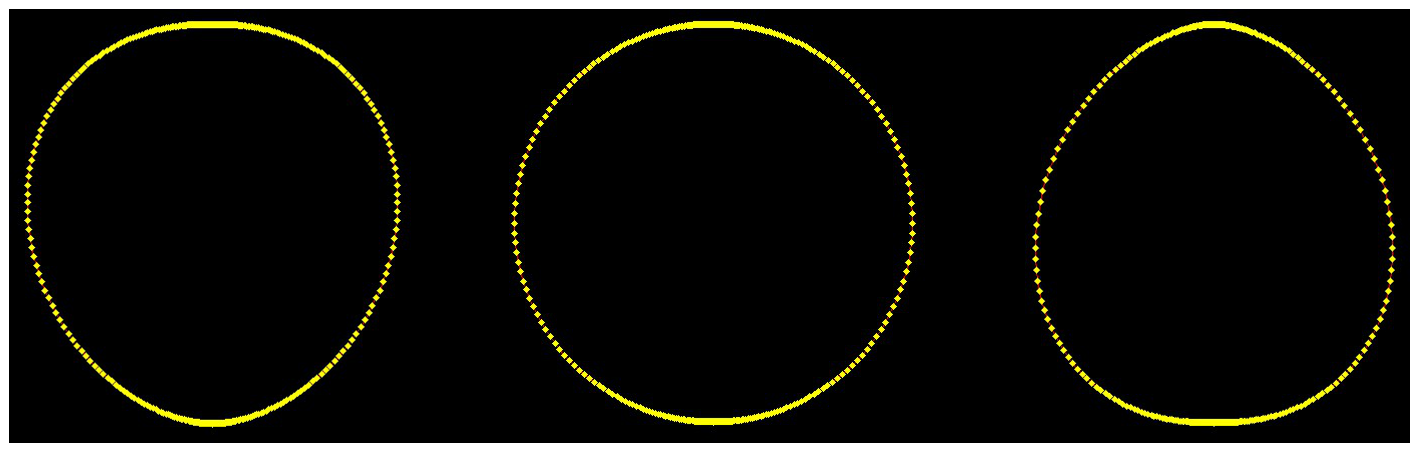
Path curves with different λ-values (left: 0.8, middle: 1.0, right: 1.3). This is the approximate range in which mistletoe berries change their shape.

Lambda (λ) is one of the shape-defining parameters in a three-dimensional path curve. It determines the outline, i.e. the two-dimensional contour of a path curve. The numerical value “1” is an equilibrium between the two variations of “egg-shapes”, meaning it has the same acumination or flatness on both of its ends. For λ ≥ 1 the top is sharper and the bottom flatter. For 0 < λ ≤1 the path curve will be the opposite, sharp at the bottom and flat at its top (see Figure 6). λ describes the relation between the shape of the path curve's top and bottom. λ is not an absolute value describing a certain shape: the λ-value can stay the same while height or width of the path curve changes. For λ < 0 the path curves transform into vortex-like forms. Figure 6 gives an impression of different λ-values as present in mistletoe berries.

LambdaFit produces a path curve arising from preset parameters such as width, height and λ. These values can also be manipulated manually (see Figure 5). If not manually set, the software uses the preset values as determined in the opening window (as shown in Figure 1). The position of the drawn path curve within the photograph can be changed by rotation and translation on the x- and y- axis. Again, the software draws the position of the path curve according to the modified values.

**Figure 7:**
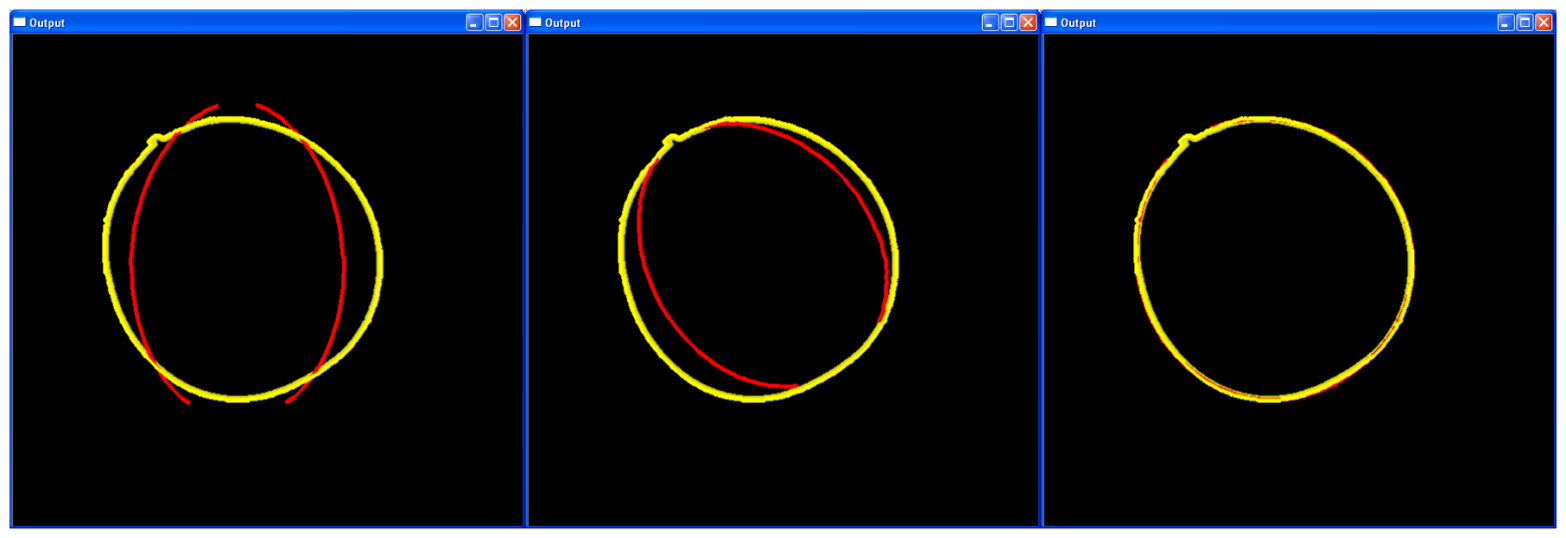
“Output” window showing the visual optimization process of a mistletoe berry's contour. Left: composite picture of a mistletoe berry with the best fitting contour in yellow and the calculated – not yet fitted – path curve in red; center: the path curve being positioned after using the hot-key “M” to define the top and bottom of the path curve; right: the path curve after the optimization using the hot-key “P”.

In order to obtain an optimal fit, it is necessary to place the starting path curve at a reasonable position. This step is critical. The operator has to define the line of symmetry or mirror axis, i.e. the top and bottom of the path curve. The operator has the possibility of defining this position via a number of “hot-keys” in combination with the cursor (computer mouse), for example, by pressing the hot-key “M” to define the mirror axis. “M” has to be pressed, then the cursor must be moved to the corresponding points: top first, and clicking, sets the position; then bottom, and clicking again, sets the axis point at the base of the object within the photograph (see Figure 7 middle). This choice of an adapted starting position will make the path curve fitting process considerably faster, since the overlapping of the contour (yellow line) and the path curve (red line) is already quite good. Figure 7 (right) shows an optimized path curve. The hot-keys “P” and “O” are available to start the curve fitting process. “P” optimizes the path curve by finding the best congruency with all parameters (see details in Design and Implementation), and “O” starts an optimization process that mainly considers the width of the path curve.

**Figure 8:**
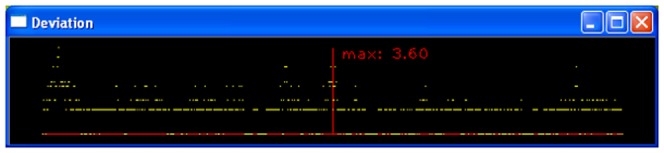
“Deviation” window. The distance in pixels is shown for every point (in pixels) along the path curves lambda line (red) to the berries outline shape (yellow). In this case the congruency is quite high. In almost all the events the distance is “0” or “1” pixel difference; toward the bottom of the berry (both ends of the lines), congruency deteriorates however to a maximum of 3.6 pixels distance on the left side.

An additional window facilitates the optical differentiation of the congruency between the real berry's outline shape (the yellow line) and the mathematical path curve, respectively the λ-line (red). This is a helpful tool, since overlapping is often so close that it is impossible to judge by eye whether further optimization is possible (as shown in figure 7 right). The “deviation” window transfers the two curved lines from the output window into straight lines and automatically translates the scale to a maximum difference between them. This presents the user with more information instead of only the average, stored distance. The distance between the lines is shown in pixel units starting from the top (center in figure 8) to bottom (far ends on both sides in figure 8) of the object i.e. berry. This enables the user to alter the variables by hand via the sliders (see figure 5) because the optimization algorithm is searching for a global minimum and often “ignores” local noticeable problems, which the deviation window makes visible (see “Fitting the path curve” in the “Design and Implementation” section). Figure 8 demonstrates the situation of an unequally fitted path curve. On the far left side (meaning, near the bottom of the berry's left side) the congruency is not as good as may be possible (there is 3.6 pixels difference between the mathematical path curve and the biological shape instead of 1 pixel distance, as this is the difference in most of the other regions of the berry's outline (dominant yellow straight line in figure 8)).

### Storing the results

Results (i.e. path curve parameters, energy function value and the time the measurement was performed) are stored in a result file. Accompanying data in this file are the real names of the images of each measurement and the time the image was taken. The latter is automatically extracted from the JPEG exif data.

The file saving the measurements is a comma-separated value file (csv) and can be opened and processed by any statistical or spreadsheet application.

### Internal validity

**Figure 9:**
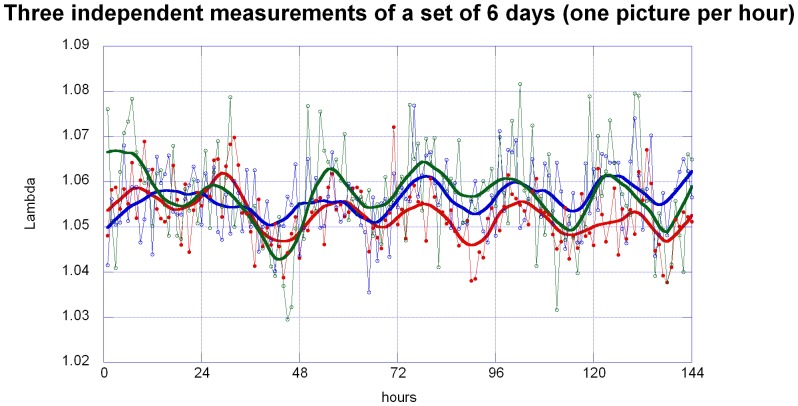
Three independent measurements of Lambda (P1, P2, P3), based on 144 pictures of the same mistletoe berry (24 pictures per day in the course of six days). P1 (red dots) and P2 (blue circles) are two independent measures by the same person, P3 (green circles) by a different person. Every data point represents the mean of several measurements (n = 10 for P1 and P3, n = 6 for P2). Error bars were omitted for clarity. The smooth continuous lines represent LOWESS fits with P = 10% (red: P1, blue: P2, green: P3).

The quality of the extracted data is sensitive to the operator's intervention at two points: the identification of the contour, and the fitting of the path curve, respectively. Thus, the necessary and sufficient number of replicate measurements per image had to be determined. Three independent evaluation runs were performed on a set of 144 pictures. They were obtained from a single berry on a mistletoe bush (*Viscum album* ssp. *album*) growing on oak (*Quercus robur*), located in Arlesheim, Switzerland. The berry was photographed from a fixed distance and angle every hour for six days in October 2011. Former observations led us to expect a circadian rhythm in the changes to the berry's shape (see Figure 9).

Two questions had to be answered: first, is it possible to reproduce one's own measurements? For this setting the same person repeated the identical set again after a longer period of time (P1 and P2 is the same person). The second question was: is it possible for a different operator to achieve the same results? In this setting a different person P3 did the computation. All photos were measured in random order as described above. To minimize random error, each photo was measured repeatedly. P1 and P3 had 10 repeats per photo; P2 had 6 repeats per photo.

Figure 9 displays the mean λ value of all repeats per photo. Additionally, a weighted fit has been added because it reveals the signal within the data, making the rhythmic change of λ visible at first sight. The software KaleidaGraph 4.0 (Synergy Software, Reading PA) was used to draw a weighted fit using LOWESS, locally weighted regression scatter plot smoothing [13]. For each data point this operation defines a linear regression equation, in which a certain percentage P of the neighboring weighted data points is included. The function thus calculates a smoothed curve; the larger the percentage P of included neighboring data points, the smoother the curve.

**Figure 10:**
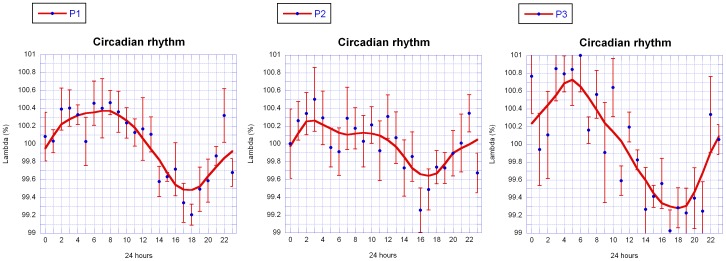
Daily change of Lambda of a mistletoe berry (mean ± SE) for three independent measurements (P1, P2, P3). Data from Fig. 9 were normalized for each day and averaged. P1 and P2 are two independent measures by the same person, P3 are measurements by a different person. The smooth continuous lines are LOWESS fits (P = 30%).

In Figure 9 an approximately circadian rhythm could be identified for P1, P2 and P3. The λ data were thus compiled on a 24 h scale to compare the measurements in more detail. First, the λ values were normalized per day (mean set to 100%). Second, mean values were calculated for each hour of the day, based on the data obtained on the six days. These values were plotted together with standard errors (Figure 10).

A LOWESS fit (P = 30%) reveals a comparable signal within the data: λ exhibits a circadian rhythm of≈0.5%. It is above the average between 0.00–12.00 am, and below average from noon to midnight. Minima and maxima vary to some extent between operators and repetition of the measurements.

Daily change data of the three measurement series, the hourly mean values (blue points in Figure 10) as well as the data of the weighted fit in hourly resolution (violet lines in Figure 10), were correlated (see Table 1) between P1, P2 and P3. For the correlation analysis we used the Pearson product moment correlation test with Statistica 6.0 (StatSoft, Tulsa, USA).

**Table 1:** Pearson correlation coefficients r of circadian rhythms measured by different operators (P1, P2, P3).

Correlation	Hourly mean		LOWESS 30% smoothed data	
	r	p	r	p
P1 to P2	0.7637	<0.0001	0.9226	<0.0001
P1 to P3	0.7864	<0.0001	0.9399	<0.0001
P2 to P3	0.6005	0.0019	0.9220	<0.0001

Correlation calculation is based on n = 24 data points each. P-values (p) are calculated by the Pearson product moment correlation test.

Comparison of the hourly mean values of P1 with those of P2 yields a highly significant correlation coefficient (p < 0.0001, n  =  24). The same applies to the data of the weighted fit (p < 0.0001, n  =  24).

The correlation analysis for P2 and P3 gives a value of the correlation coefficient of r  =  0.60 (p  =  0.0019, n  =  24), which is less pronounced than the correlation between P1 and P3 (r  =  0.79). The reason for this is most probably of technical nature, since the data for the circadian rhythm of P2 were compiled from six repeated Lambda determinations (instead of 10 for P1 and P3). For the future, means of 10 repeated Lambda determinations per photograph will be used.

The significant correlations show that both the handling of the data and the high-level functionality of the software developed will allow robust operation.

## Design and Implementation

### Drawing the path curve

The function used in drawing a 2-dimensional path curve contour is:
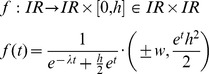



This function can be derived by using the continuous transformation matrix
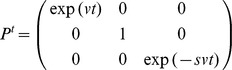
for a plane path curve with three real and different eigenvalues with v  =  1 and s  =  λ [14, p. 20].

On the other side it is possible to derive the path curve funktion *f* starting from the equations (14) and (15) from Almon [6]:
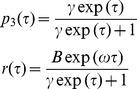



Almon is using as independent variable τ for *p*_3_(τ) and t = τ for *r*(*t*). In order to be unambigous, we continue to use τ as independent variable, which corresponds to equation (15) of Almon.

Substituting τ, γ, *B* and ω according to
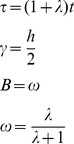
one gets the path curve equation needed for the software:
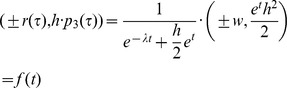



The ± sign in the x-coordinate indicates that f draws the right and the left side of the path curve simultaneously. Multiplication of *p*_3_(τ) with *h* in the *y*-coordinate is needed to get an arbitrary height.The limit values for the domain *IR* are *f* (–∞) = (0,0) and *f* (∞) = (0,*h*):
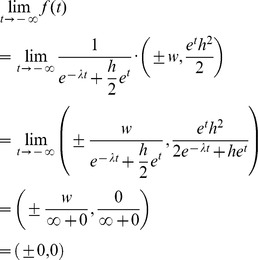


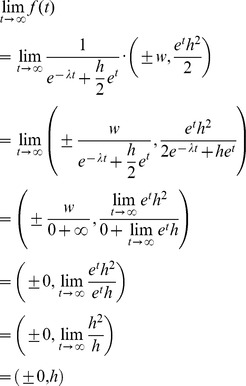



Thus, h can be considered the parameter defining the path curve's height. The other two constants in f are w and λ. w can be considered the parameter defining the path curve's width, as it is simply a factor for the curve's y-coordinates. The most interesting parameter is λ, which defines the characteristic form of the curve; pointed top, round or pointed bottom (see Figure 6).

**Figure 11:**
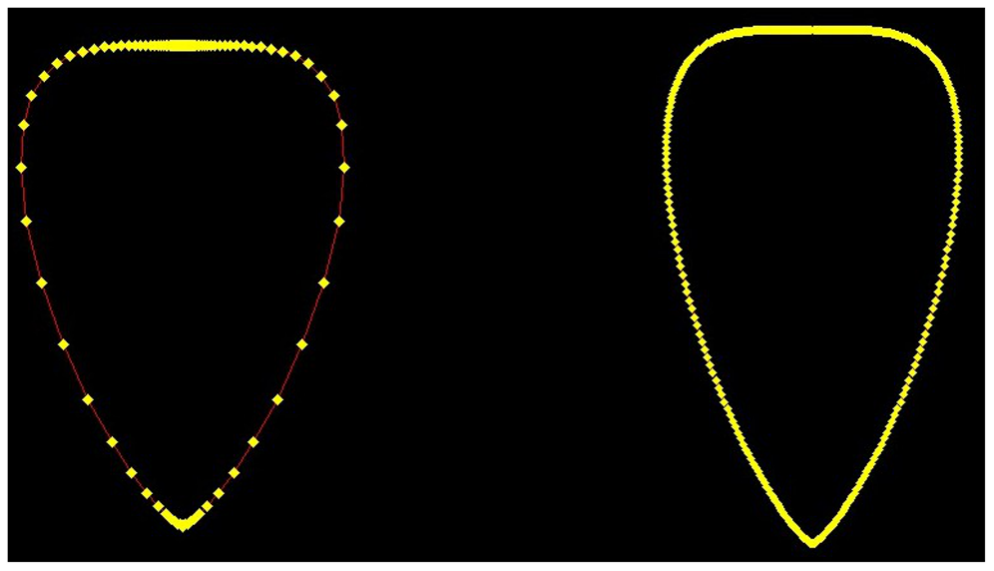
The g(t)-points of a path curve (λ = 0.3), with *t* ∈ [−20, 20]. Left: with a step size of 0.4: *t* = 0.4*·i, i* ∈ *IN*. Right: with a step size of 0.05: *t* = 0.05*·i, i* ∈ *IN.*

The task for our software is to find the parameters h, w and λ, so that the resulting path curve has an optimal congruence with the measured plant contour from the picture. The measured contour can, however, be located and oriented anywhere in the photograph. So we also need rotation, horizontal and vertical translation as parameters α, x and y. Adding these to f, we get:
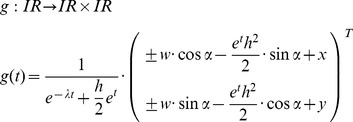



With this set of six parameters the program is able to draw every possible path curve, showing always only the two-dimensional λ-shape. In the next step, we have to decide how many points of g we want to calculate. As the domain of t is *IR*, the path curve consists of an infinite number of points. For equidistant t values however, the resulting points g(t) are found to lie more densely toward the top and bottom of the path curve (Fig. 11).

It is therefore sufficient to calculate values from t = −20 to t = 20. To obtain enough intermediate points we chose a step size of 0.05 between the different t values, which led to a total of 801 calculated points for each side (left and right) of the path curve.

As shown in figure 11, the calculated points are connected with straight red lines. Thus, we can get all pixel coordinates of the calculated path curve, including the points between the ones calculated by g(t). If the step size between subsequent values of t is sufficiently small – which is the case for 0.05 – the gaps between the calculated points are so small that the path curve seems to exhibit a round shape, even if single points are connected by straight lines. Thus the calculated path curve has no continuous curvature. This deviation from an ideal path curve is negligible, however, compared with the photographs' resolution, and naturally occurring variations in biological shape.

### Fitting the path curve

The software has to bring the pixels of the measured plant contour and the pixels of the calculated path curve into congruence by finding the optimal parameters λ, h, w, α, x and y. This is achieved by a so-called hill-climbing algorithm. We start with an arbitrary path curve and check how congruent it is with the plant contour. Then we change its parameters and check again. If the new parameters yield a more congruent path curve we keep the new ones, and repeat the procedure. Eventually the best fitting path curve is found. We will discuss the details of this algorithm below. Before this, we describe how the software checks the degree of congruence of a calculated path curve with the measured plant contour.

Congruency of the two curves is estimated by a so-called energy function, as input of the two contours. A lower value is returned if these are more congruent, and a higher value if they are less congruent. To achieve this, we perform a distance transformation on the image of the measured plant contour.

**Figure 12:**
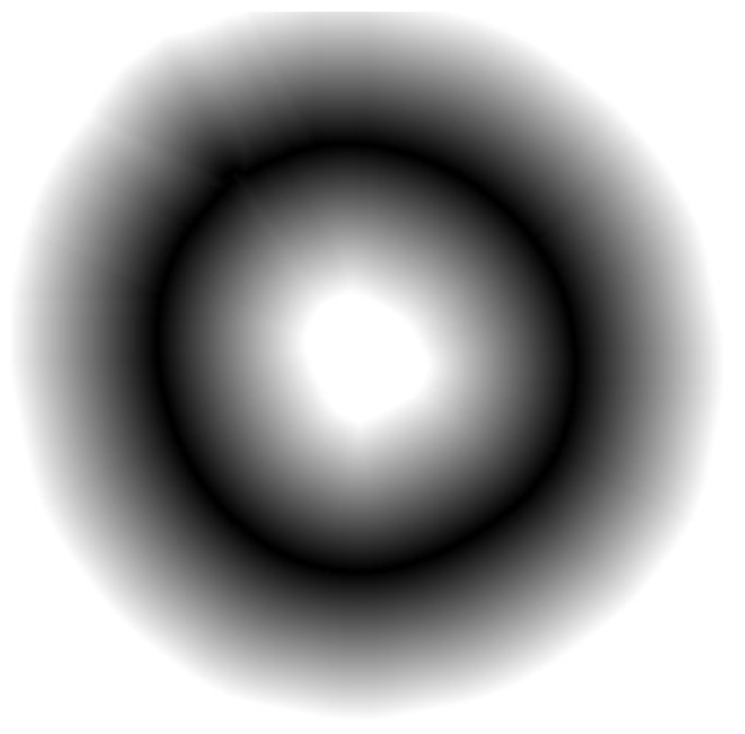
Plant contour from Fig. 3 after distance transformation. Each pixel coordinate holds as its value the Euclidean distance between the pixel itself and the closest pixel of the plant contour. The darker a pixel, the closer it is to the plant contour.

The distance transformation can be done by another method of openCV [15]. After the distance transformation of a plant contour image (see figure 2 for an example), the newly generated image contains for each pixel the Euclidian distance of this pixel from the nearest plant contour pixel (see Figure 12).

By means of this distance transformation we can immediately determine the distance of each pixel in the calculated path curve from the measured plant contour by checking the values of each pixel in the distance transformation image with the coordinates of the pixels in the path curve. The greater the distance between the calculated path curve and the plant contour, the higher is the sum of all these values. Thus it makes sense to define this sum as energy function. The perfectly fitted path curve would produce an energy function value of 0, because every pixel lies on the measured plant contour.

Letting n be the number of all pixels in the calculated path curve, and letting dist_i_ be the value of the coordinates belonging to pixel i in the distance transformation image, our energy function could be defined as:
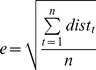



To allow statistical interpretation, we modified this energy function by summing the squares of the distances, and thus essentially arrived at the usual least-squares fitting procedure: 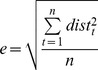



With the energy function defined as above, we can come back to the hill-climbing algorithm. As mentioned above, the algorithm can start with an arbitrary path curve. It then alters some (or all) of the six parameters of g(t), which define the path curve, by a certain proportion δ and checks if these changes yield a path curve more congruent with the plant contour. If so, the new parameter values are kept and the procedure starts again. If no better path curve can be found, the algorithm does not stop but decreases δ. Thus, the longer the search lasts, the more fine-tuned it becomes. If δ reaches a certain minimal value, the algorithm stops and a minimum of the energy function is found. The following pseudo code illustrates this:

*curve*: = *initial*_*curve*;

*δ*: = 0.5;

while (δ >0.005) {

*found*_*better*: = true;
while (*found*_*better*) {
    
*found_better*: = false;
    “Alter the parameters of *curve* by *δ*
    (several alterations are possible)”
    if (*alteration_k* is better than *curve*) {
       
*curve*: = *alteration*_*k*;
       
*found_better*: = true;
    }
}
*δ*: = *δ*/1.6;

}

Of course, this hill-climbing algorithm is prone to not finding the global minimum, but only a local minimum of the energy function. To overcome this, the human software operator has to supervise the algorithm by means of a graphic user interface, which shows the original plant image, the recognized plant contour and the calculated path curve in one image (see Figure 4).

### Streamlining the optimization process

In Figure 4 it can be seen that the top and bottom of the calculated path curve are excluded. This “cut off” functionality was integrated because of the actual habitus of the plant. The bottom parts of mistletoe berries deviate from ideal path curves due to their fixation at the stipe (peduncle). Correspondingly, the berry-shape is not always clearly identifiable by the software. Likewise, the little cap on top of the berries does not belong to the path curve shape of the berry. In order not to let these irregularities have an impact on the energy function, we can set top and bottom of the path curves for exclusion from the measurement.

Another peculiarity of mistletoe berries is that they are almost round. This makes it almost impossible for the hill-climbing algorithm to find the correct rotation of the path curve. We therefore enabled the human operator to define the coordinates of the top and bottom of the path curve by clicking at the positions in the image. The algorithm then runs in a mode where no greater variations of the rotation are allowed, because the orientation set by the operator is maintained.

We also found that the hill-climbing algorithm works particularly well if we first let it optimize only the width parameter w of the path curve, followed by an optimization cycle for the λ parameter only, followed by a last cycle in which again variations of all six parameters were allowed.

To enhance the optimization we also applied slight changes to the path curve-drawing function g(t),. One change was that an alteration of α would lead to a rotation of the path curve around its center, instead of around its top, which was the case for the original g(t) function. Another change was that we always multiply the current parameter value of w with the current value of λ, and use this product for w. This compensates for the overall effect which an alteration of λ has on the path curve's width. With this change, an alteration of λ mainly affects the bow of the curve rather than its width.

## Conclusion and Future Work

The program may be improved by future users so as to offer more pre-set versions of the variable values at the starting process when importing a file, to accommodate the precise indicator of phenotypical change of shape in berries or buds. A circadian rhythm in mistletoe berries has been found and will be investigated further. Different, long-frequency rhythms will be studied as well. The software will be applied to study of the leaf buds of mistletoe host trees, in order to search for interactions in rhythm. Corresponding rhythms or rhythmical interactions of mistletoe berries and the leaf buds of mistletoe host trees will give valuable insights into the biology of parasitism, i.e. mutualism in plant biology.

### Availability and future directions

The software called LambdaFit is available along with further directions and bud and mistletoe berry samples at http://www.feiten.de/LambdaFit. We are also offering its source code for free download and further development together with compilation instructions. More information on the libraries used, and their availability, are included on the website as well.
